# Deutlicher Überlebensvorteil durch simultane Radio- und Chemotherapie: Erkenntnisse aus einer chinesischen Phase-III-Studie bei älteren Patienten mit Ösophaguskarzinom

**DOI:** 10.1007/s00066-022-01921-6

**Published:** 2022-03-14

**Authors:** Claudia Schweizer, Rainer Fietkau, Florian Putz

**Affiliations:** Strahlenklinik, Friedrich-Alexander-Universität Erlangen-Nürnberg, Universitätsstr. 27, 91054 Erlangen, Deutschland

## Hintergrund und Ziel der Arbeit

Nicht nur angesichts der demografischen Veränderungen ist die Entwicklung von geeigneten radioonkologischen Therapiekonzepten für ältere Patienten sehr wichtig. Beim Ösophaguskarzinom ist beim betagten Patienten in der definitiven Situation die Verträglichkeit üblicher simultaner Kombinationschemotherapien häufig schlechter und deren Stellenwert daher umstritten. Die Autoren Ji et al. verglichen nun in einer randomisierten Phase-III-Studie eine simultane Radiochemotherapie (RCT) mit dem oralen Fluorouracilderivat S‑1 gegenüber einer alleinigen Radiotherapie (RT) bei betagten Patienten mit Ösophaguskarzinom.

## Methode

In dieser randomisierten, unverblindeten, multizentrischen, chinesischen Phase-III-Studie wurden ältere Patienten im Alter von 70 bis 85 Jahren mit histologisch gesichertem Ösophaguskarzinom im Stadium IB bis IVB und ECOG-Status ≤ 1 eingeschlossen. Stratifiziert wurde nach Alter (< 80 vs. ≥ 80 Jahre) und Tumorlänge (< 5 vs. ≥ 5 cm) bei einer 1:1-Randomisation zwischen alleiniger definitiver RT und einer simultanen RCT mit dem oral applizierbaren Chemotherapeutikum S‑1. Primärer Endpunkt war das 2‑Jahres-Gesamtüberleben (OS).

## Ergebnisse

Von 2016 bis 2018 wurden 298 Patienten aus 23 chinesischen Zentren eingeschlossen. Das mediane Alter betrug 77 Jahre und praktisch alle Patienten wiesen histologisch ein Plattenepithelkarzinom auf (99,3 %). In Bezug auf die Therapiedurchführbarkeit war der Anteil an Therapieabbrüchen mit 22,1 % im RCT-Arm gegenüber nur 10,1 % im RT-Arm deutlich höher, die Therapie also schwieriger durchzuführen. Bei den höhergradigen Toxizitäten (≥ Grad 3) war einzig das Auftreten von Leukopenien im Kombinationsarm signifikant erhöht (9,5 % vs. 2,7 %), und therapieassoziierte Todesfälle traten nur bei 3 Patienten (2,0 %) im Kombinationsarm auf im Vergleich mit 4 Patienten (2,7 %) bei alleiniger Radiotherapie. Das 2‑Jahres-Gesamtüberleben, der primäre Endpunkt der Studie, war bei simultaner Radiochemotherapie signifikant verbessert (53,2 % vs. 35,8 %, *p* = 0,002).

## Schlussfolgerung der Autoren

Die simultane Radiochemotherapie mit S‑1 war verträglich und bringt einen signifikanten Überlebensvorteil gegenüber einer alleinigen Strahlentherapie bei älteren Patientinnen und Patienten mit Ösophaguskarzinom.

## Kommentar

Fast genau 30 Jahre, nachdem Herskovic et al. eindrucksvoll den therapeutischen Gewinn durch eine simultan zur definitiven Strahlentherapie des Ösophaguskarzinoms durchgeführten Chemotherapie demonstrieren konnten [1], zeigten nun Ji et al. erneut, dass das Paradigma der simultanen RCT auch beim älteren Patienten mit Plattenepithelkarzinom des Ösophagus bemerkenswert gültig ist [2].

In der von Ji et al. betrachteten, generell herausfordernden Patientenpopulation älterer Patienten mit Ösophaguskarzinom ist sonst die Durchführbarkeit [3–5] und Verträglichkeit [1, 6, 7] der üblichen simultanen Kombinationsschemata deutlich schlechter, und ein prognostischer Vorteil der simultanen RCT stand teilweise im Zweifel [8]. Man muss sich nämlich bewusstmachen, dass gerade für diese Gruppe der älteren Patienten mit Ösophaguskarzinom die Erwartung an eine optimale definitive radioonkologische Behandlung besonders hoch ist. Denn häufig besteht ja kurative Inoperabilität.

Durch Phase-I- und -II-Studien vorbereitet setzten die Autoren auf eine radiosensitivierende simultane Monochemotherapie [9, 10]. S‑1 (Handelsname Teysuno®) ist ein vor allem in Asien beliebtes orales Kombinationspräparat aus der 5‑FU-Prodrug Tegafur und den zwei modulierenden Substanzen Gimeracil und Oteracil. Gimeracil ist dabei ein reversibler Inhibitor der Dihydropyrimidin-Dehydrogenase (DPD), der die Halbwertszeit von 5‑FU erhöht, und Oteracil inhibiert durch seine geringe orale Bioverfügbarkeit im Magen-Darm-Trakt selektiv das aktivierende Enzym Orotat-Phosphoribosyl-Transferase, was die gastrointestinalen Nebenwirkungen reduzieren soll [11]. S‑1 ist generell auch in Europa, nicht aber in den USA zugelassen und damit auch in Deutschland erhältlich. In der hier kommentierten Studie hatten die Patienten im Kombinationsarm die orale Chemotherapie mit S‑1 jeweils in der 1. und 2. sowie 5. und 6. Woche der 6‑wöchigen simultanen RCT eingenommen. Die Verträglichkeit der simultanen Chemotherapie war insgesamt gut. Insbesondere gab es keine Häufung von höhergradigen Ösophagitiden und anderen gastrointestinalen Nebenwirkungen und keine Häufung von Infektionen bzw. therapiebedingten Todesfällen. Lediglich die höhergradigen Leukopenien waren statistisch signifikant unterschiedlich, aber lediglich geringfügig von 2,7 % auf 9,5 % erhöht (*p* = 0,01). Die Therapiedurchführbarkeit im Kombinationsarm mit einer Abbruchrate von 22,1 % vs. 10,1 % im alleinigen RT-Arm kann in diesem Kollektiv noch als zufriedenstellend angesehen werden. Analog zu der grundlegenden Arbeit von Herskovic et al. erhielten die Patienten im Kombinationsarm eine geringere Radiotherapiedosis mit 1,8 Gy bis insgesamt 54 Gy, verglichen mit 2 bis 60 Gy bei alleiniger RT (2 bis 50 Gy vs. 2 bis 64 Gy in der Herskovic-Arbeit). Das klinische Zielvolumenkonzept entsprach dabei weitgehend den üblichen Standards mit einer kraniokaudalen Erweiterung von jeweils 3 cm und einem zirkumferenziellen Sicherheitssaum von 1 cm für den Primärtumor sowie von 0,5 bis 1 cm für die bildmorphologisch befallenen Lymphknoten bei einem PTV-Sicherheitssaum von 0,5 bis 1 cm. Interessanterweise konnten Patienten im Alter von 70 bis 79 Jahren zusätzlich eine elektive Bestrahlung der Lymphknotengruppen erhalten. Eine FDG-PET-CT war für die Zielvolumendefinition hilfreich, jedoch nicht verpflichtend [12].

Umso verblüffender erscheint uns der ganz erhebliche Wirkungsgewinn, der durch eine so milde Radiosensitivierung erreicht werden konnte: Die klinische Komplettremission verbesserte sich beispielsweise von 26,8 % auf 41,6 % (*p* = 0,007), das mediane Progressionsfreies Überleben (PFS) von 9,5 auf 18,7 Monate (*p* = 0,003) und das OS in der Intention-to-treat-Analyse nach 2 Jahren von 35,8 % auf 53,2 % mit Reduktion der Hazard Ratio auf 0,63 (*p* = 0,002). Interessanterweise zeigte die Subgruppenanalyse, dass auch Patienten mit ≥ 80 Jahren signifikant von der simultanen Chemotherapie profitierten (HR 0,56, 95 %-CI 0,33–0,98).

Eine wichtige Einschränkung der Aussagekraft ist, dass nahezu ausschließlich Patienten mit Plattenepithelkarzinomen behandelt wurden (99,3 %). Eine direkte Übertragung der Ergebnisse auf Patienten mit Adenokarzinomen ist daher leider nicht möglich, zumal diese bekanntermaßen beim Ösophaguskarzinom anders auf eine simultane RCT ansprechen als Plattenepithelkarzinome [13]. Und da lediglich ältere Patienten mit einem ECOG von 0 bis 1 eingeschlossen wurden, ist der therapeutische Vorteil für ältere Patienten im schlechten Allgemeinzustand auch nicht zu beurteilen. Am anderen Ende des großen Spektrums an physiologischen Leistungsreserven, die wir in der klinischen Praxis bei älteren Patienten sehen, gibt es sicherlich auch fitte 70-Jährige, denen man eher eine simultane RCT angedeihen lässt als eine Monochemotherapie entsprechend dem bisherigen Standard. Es sind daher wie so oft bei älteren Patienten auch der sog. ärztliche Blick des Behandlers und die subjektive Einschätzung des biologischen Patientenalters, die die Therapieauswahl bestimmen [14].

## Fazit

Die hier kommentierte Phase-III-Studie zeigt, dass eine simultane definitive RCT mit S‑1-Unterstützung auch von älteren Patienten mit Plattenepithelkarzinomen des Ösophagus gut vertragen wird und auch bei sehr alten Patienten mit 70 bis 80 Jahren signifikant und deutlich das Gesamtüberleben verbessert. Eine simultane Radiochemotherapie mit S‑1 stellt daher beim älteren Patienten mit > 70 Jahren und einem Plattenepithelkarzinom des Ösophagus den neuen Standard für die definitive radioonkologische Behandlung dar. Darüber hinaus zeigen die Ergebnisse der Studie auch, dass das Paradigma der simultanen RCT 30 Jahre nach seiner Erstpublikation durch Herskovic et al. nach wie vor auch für ältere Patienten mit Ösophaguskarzinom eindrucksvolle Gültigkeit besitzt.

*Claudia Schweizer, Rainer Fietkau*

*und Florian Putz, Erlangen*
